# Short term outcome of laparoscopic ventral rectopexy for rectal prolapse

**DOI:** 10.12669/pjms.324.10196

**Published:** 2016

**Authors:** Muhammad Naeem, Mariyah Anwer, Muhammad Shamim Qureshi

**Affiliations:** 1 Dr. Muhammad Naeem, Senior Registrar General Surgery, Department of Surgery, Ward 2, Jinnah Postgraduate Medical Centre, Karachi, Pakistan; 2 Dr. Mariyah Anwer, Senior Registrar General Surgery, Department of Surgery, Ward 2, Jinnah Postgraduate Medical Centre, Karachi, Pakistan; 3 Muhammad Shamim Qureshi, Professor of General Surgery, Department of Surgery, Ward 2, Jinnah Postgraduate Medical Centre, Karachi, Pakistan

**Keywords:** Ventral rectopexy, Laparoscopic nerve sparing ventral rectopexy (LVR), Complete rectal prolapse management

## Abstract

**Objective:**

To find out the short term outcomes of effectiveness and safety of laparoscopic ventral rectopexy for rectal prolapse.

**Methods:**

It was a descriptive case series study of 31 consecutive patients of rectal prolapse in Colorectal division of Ward 2, Department of General surgery, Jinnah Post Graduate Medical Center, Karachi, from November 2009 to November 2015. These patients were admitted through outpatient department with complains of something coming out of anus, constipation and per rectal bleeding. All patients were clinically examined and baseline investigations were done. All patients underwent laparoscopic repair with ventral mesh placement on rectum.

**Results:**

Among 31 patients, mean age was 45 years range (20 - 72). While females were 14(45%) and males 17(55%). We observed variety of presentations, including solitary rectal ulcers (n=4) and rectocele (n=3) but full thickness rectal prolapse was predominant(n=24). All patients had laparoscopic repair with mesh placement. Average hospital stay was three days. Out of 31 patients, there was one (3.2%) recurrence. Port site minor infection in 3(9.7%) patients, while conversion to open approach was done in two (6.4%), postoperative ileus observed in two (6.4%) patients. one(3.2%) patient developed intractable back pain and mesh was removed six weeks after the operation. one(4.8%) patient complained of abdominal pain off and on postoperatively. No patient developed denovo or worsening constipation while constipation was improved in 21 patients (67%). Sexual dysfunction such as dysperunia in females and impotence in males was not detected in follow up.

**Conclusions:**

This study provides the limited evidence that nerve sparing laparoscopic ventral rectopexy is safe and effective treatment of external and symptomatic internal rectal prolapse. It has better cosmetic and functional outcome as advantages of minimal access and comparable recurrence rate.

## INTRODUCTION

Internal and external rectal prolapse are not life-threatening conditions, these disorders can be extremely debilitating and have a negative impact on quality of life.^1^ Rectal prolapse is associated with fecall incontinence and constipation in majority of patients.^2^ Affected individuals with rectal proplapse may report discomfort or pain from prolapsing tissue, drainage of mucus or blood, and associated fecal incontinence or difficult evacuation. Women aged 50 and older are 6 times more likely as men to present with rectal prolapse.^3^ Two thirds of women are multiparous and 15 to 30% are reported to have associated urinary dysfunction and vaginal prolapse

Numerous surgical procedures, both perineal and abdominal, are currently practiced for the treatment of complete rectal prolapse. The abdominal operations carry a lower recurrence rate and improved functional outcome and are therefore preferred over the perineal operations. The latter are reserved for those who are unfit to undergo an abdominal (e.g. a laparoscopic) procedure.^4^

In 2004 D Hoore introduced laparoscopic ventral rectopexy with very promising results.^5^ The unique feature of this technique is that it avoids any posterolateral dissection of the rectum in order to avoid denovo constipation and sexual dysfunction. The mesh is sutured to the anterior aspect of the rectum to inhibit intussusception. Because this technique limits the dissection and the subsequent risk of autonomic nerve damage, the functional outcome is improved with minimal long term morbidity and low rates of recurrence and the short term follow up results are very convincing.

Ventral rectopexy has gained popularity in Europe to treat full-thickness rectal external and internal prolapse Over the last decade, as for other procedures, biological meshes are used to correct rectal prolapse.^6^ **This procedure has been shown to achieve acceptable anatomic results with low recurrence rates, few complications, and improvements of both constipation and fecal incontinence.**7 **Although long term results are being assessed, learning curve affects the outcome in initial series and complications are related to the learning curve as well as the techniques.**8

## METHODS

This descriptive study comprises of 31 patients who were scheduled for laparoscopic ventral rectopexy. All patients were admitted through outpatient department of colorectal division Ward 2 JPMC, from November 2009 to November 2015. Patients with full thickness rectal prolapse, Solitary rectal ulcer syndrome with internal prolapse and rectocele were included in the study. Patients who had previous abdominal surgery or had pelvic malignancy or were not fit for General anesthesia were excluded. Most of the patients complained of something coming out of anus and bleeding per rectum. All patients were clinically examined and baseline investigations and sigmoidoscopy was done along with defecogram in selected cases. Informed Consent was taken. Permission from Hospital Ethics Committee was taken to carry out our study.

In Surgery, all patients were operated in general anesthesia on elective Operation Theatre lists. There is no lateral dissection leaving lateral ligaments intact thus preventing recurrence. Avoidance of posterior rectal mobilization leaves autonomic nreves intact thus preventing denovo constipation. Strip of non absorbable mesh is sutured to the ventral aspect of distal rectum caudally preventing intussusseption. Extraperitonealization avoids mesh related complications

The data of different variables like age, gender, postoperative hospital stay and complications including recurrence were collected on follow up. Anorectal function was recorded and Incontinence was assessed by Cleveland Clinical Score, while constipation was assessed by Rome – II criteria. Sexual Dysfunction as dyspareunia and retrograde ejaculation or potency problems were asked in follow up.

The data was collected as mean and range and assessed by SPSS v 20 and demographic variables were analysed and t-test applied on complications, p value < 0.05 was considered statistically significant.

## RESULTS

There were total 31 patients of laparoscopic ventral rectopexy, in which 14 were females (45%) and 17 were males (55%) Fig.I Mean age was 43.55 years (range 17-81 years)

**Fig-I:**
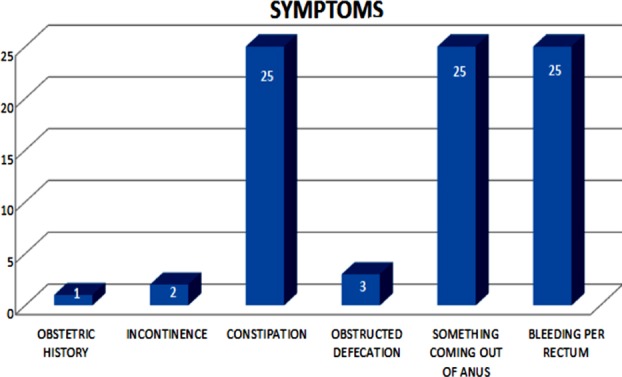
Clinical presentation.

Most of our patients presented with symptoms of something coming out of anus, bleeding per rectum and constipation i.e. 25(81%), while incontinence was present in 2(6.4%), obstructed defecation in 3(9.7%) and obstetric history in 1(3.2%) patient. Fig.II

**Fig-II:**
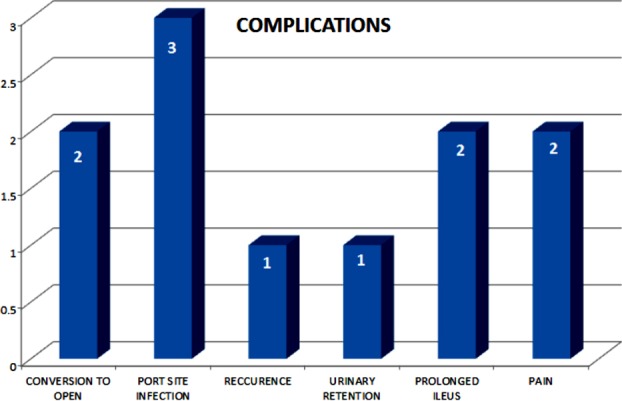
Clinical presentation.

There were three main indications for this procedure. In majority of patients, operation was done for full thickness rectal prolapse, 24(77.4%), followed by solitary rectal ulcer syndrome with internal prolapse, n=4 (13%) and 3 (9.7%) cases of rectocele

Mean operating time was 150 minutes while 2(6.4%) cases were converted into open operation due to bladder injury in one patient and one patient had narrow pelvis resulting in difficult surgical access. Mean time for bowel function return was 24 hours. Mean hospital stay was three days (range 2 -11 days). In early Postoperative phase urinary retention was seen in 1(3.2%) patient and port site infection in 3(9.7%) patients.

Patients were followed for complications for 6 to 18 months. Recurrence was seen in one (3.2%) patient after 6 months. Prolonged ileus were seen in 2(6.4%) patient each. One patient had repeated abdominal pain off and on but all his investigations were normal so we suspected adhesion of gut. One patient developed intractable backache and was referred to pain clinic but pain treatment failed and mesh was removed from sacral promontry laparoscopically after two months. None of the patient developed denovo constipation. A significant reduction in symptoms of constipation was reported (81% of patients before vs 13% after surgery, P < 0.001).

## DISCUSSION

The present study represents our initial experience with this newer technique of the autonomic nerve sparing laparoscopic ventral rectopexy for rectal prolapse repair. Complete rectal prolapse is a debilitating condition, which affects both the very young and elderly people and can cause fecal incontinence. The cause of the disease is unknown, but anatomical disturbances are commonly found.^9^,^10^

D Hoore in 2004 popularized a novel approach of laparoscopic autonomic nerve sparing rectopexy for complete rectal prolapse.^11^,^12^ The main objective of the treatment is to correct the anatomical defect, alleviate bowel dysfunction and avoidance of functional abnormalities of incontinence, constipation, and pain, with an acceptable rate of recurrence and the lowest rate of complications. Abdominal rectopexy is preferred method in controlling rectal prolapse.^13^ The recurrence rate varies from 0- 16% which may be due to the variation in operative technique and difference in the length of follow up.^14^

Laparoscopic rectopexy has been demonstrated to be superior to its open counterpart with recurrence rate in initial data of 5%^15^ but later with different types of meshes it is between 12 to 21%.^16^ One recurrence in the present series in the early post operative phase reflects the learning curve of the procedure, the mesh in this case was more straight resulting in the possible detachment from sacral promontry.

The identified causes of rectopexy failures are inadequate ventral dissection, improper fixation of the mesh to the anterior rectal wall, detachment of the mesh from sacral promontory, wrongly positioned staples to the upper sacrum and improper fixation of the mesh to the right rectal wall.^17^

Post operative constipation is a serious concern after abdominal rectopexy seen in 14.4% of the patient, which is mainly due to deep lateral dissection interfering with the extrinsic sympathetic innervations.^18^ Constipation was one of the leading presentations in our series. This was improved upto 67% in the short term follow up. Full mobilization may cause autonomic nerve damage disturbing recto sigmoid motility apart from neurological causes. Redundant or kinked sigmoid colon folds over the fixation area and delays transit time. Similarly rectal wall edema due to mobilization is one of the contributory factors. We experienced no denovo constipation in our limited series because no postero lateral mobilization is required and dissection is limited to the anterior aspect of the rectum in the ventral rectopexy.^19^

One of the challenging issues during the rectal mobilization in rectopexy is the damage to the autonomic nerve during rectopexy in the young sexually active patients resulting in sexual impairment and dyspaerunia. We have not encountered this problem in our short term follow up which is due to the avoidance of posterior dissection and leaving Denonvilliers fascia intact.^20^

Obstructed defecation was one of the presentations in our series. Considering the defecographic data it is shown that rectorectal intususseption is the leading cause of external prolapse.^18^ Similarly patients with solitary rectal ulcer syndrome predominantly have prolapse of the anterior wall. This prolapse is best addressed by the ventral positioning of the mesh.^21^,^22^ Rectocele also was one of the presentation of obstructed defecation syndrome without other pelvic organs prolapse.

Major mesh related complications include erosion into the vagina, bladder or rectum, mid-rectal stricture, rectovaginal fistula and chronic pelvic pain due to pudendal nerve irritation or chronic inflammation around the mesh.^23^ Mesh related complications such as pelvic abscess occurrence, erosion, and extrusion have created a doubt among the surgeons about the type of mesh used for the rectopexy. However there is no circumstantial evidence to support the use of one type of mesh over the other.^24^ The overall failure rate for biologic mesh was high upto 23% vs 9% for synthetic mesh. Synthetic mesh has the advantage of high tensile strength, immediate availability, and cost-effectiveness and tissue integration.

In our experience, the choice of the use of suture instead of staplers was based on the fact that the fixation of the meshes was safer as we take seromuscular stitches over rectum and it is easier. The use of staplers might well be an improvement in the procedure of laparoscopic anterior rectopexy to the promontory.

Dixon et al believe that learning LVMR presents two types of challenges: anatomical and technical. For trainee surgeons the anatomy and dissection planes must be learned. For experienced surgeons, transitioning to laparoscopy requires adjusting to a new perspective on pelvic and abdominal anatomy.^16^

Vertebral discitis is a rare but debilitating complication and is more frequently reported in recent studies.^25^ Intervertebral infection due to mesh was reported in one patient which gave rise to intractable pain leading to mesh removal. Due to more favorable outcomes, in selected cases this procedure can be opted as a daycare surgery in selected patients.^26^

The authors believe minimally invasive approach, modern energy devices and closure of peritoneum over the mesh reduces the risk of pelvic hematoma and subsequent infection. High tensile strength and cost effectiveness make synthetic mesh a reasonable option for rectopexy. The small number of adverse events noted so far may be the reflection of short duration of follow up. **Small sample size** is also the limitation of our study.

## CONCLUSION

**Laparoscopic ventral rectopexy not only reduces the prolapse and corrects the anatomy but also improves the function and the associated symptoms of incontinence and obstructive defecation with low rates of complication and recurrence.**
